# Physiological and transcriptome analysis of heteromorphic leaves and hydrophilic roots in response to soil drying in desert *Populus euphratica*

**DOI:** 10.1038/s41598-017-12091-2

**Published:** 2017-09-22

**Authors:** Arshad Iqbal, Tianxiang Wang, Guodong Wu, Wensi Tang, Chen Zhu, Dapeng Wang, Yi Li, Huafang Wang

**Affiliations:** 1 College of Biological Sciences and Biotechnology, National Engineering Laboratory for Tree Breeding, Beijing Forestry University, Beijing, 100083 China; 2 Department of Plant Science, University of Connecticut, Storrs, CT 06269 USA

## Abstract

*Populus euphratica* Olivier, which has been considered as a tree model for the study of higher plant response to abiotic stresses, survive in the desert ecosystem characterized by extreme drought stress. To survive in the harsh environmental condition the plant species have developed some plasticity such as the development of heteromorphic leaves and well-developed roots system. We investigated the physiological and molecular mechanisms enabling this species to cope with severe stress caused by drought. The heterophylly, evolved from linear to toothed-ovate shape, showed the significant difference in cuticle thickness, stomata densities, and sizes. Physiological parameters, SOD, POD, PPO, CAT activity, free proline, soluble protein and MDA contents fluctuated in response to soil drying. Gene expression profile of roots monitored at control and 4 moisture gradients regimes showed the up-regulation of 124, 130, 126 and 162 and down-regulation of 138, 251, 314, 168 DEGs, respectively. Xyloglucan endotransglucosylase/ hydrolase gene (*XET*) up-regulated at different moisture gradients, was cloned and expressed in tobacco. The *XET* promoter sequence harbors the drought signaling responsive *cis*-elements. The promoter expression activity varies in different organs. Over-expression and knocked down transgenic tobacco plant analysis confirmed the role of *XET* gene in roots growth and drought resistance.

## Introduction

Abiotic stress, drought stress particularly, impacts plant productivity^1^, and imposes restrictions on the distribution of plant species across different types of environments. Some plants, for example trees, have evolved distinct morphological and physiological traits^2^, and exhibit strong tolerance to water shortage than other plants^3^. The desert popular, *Populus euphratica* (Olivier), is a unique tree that can survive in the serious desert environments (Figure S1) and exhibits remarkable tolerance to environmental stresses^4^. Due to its greater ability to cope with environmental stresses, *P. euphratica* is widely considered as an ideal model system when studying the molecular mechanisms of abiotic stress responses in woody species^4–10^.

The *P. euphratica* developed heteromorphic leaves to acclimatize to the desert conditions. During developmental stages, the leaf appears in several shapes, stomata structures, epidermal appendages (wax crystals and trichomes) and specialized cells (mucilage cells and crystal idioblasts)^11^. The structural characteristics of the heteromorphic leaf are related to its eco-adaptability^12^, achieved by the differential gene expression^13^, which might alter specific regulatory pathways according to the degree of drought stress it receives^14^. Recently, transcriptome analysis of *Populus* leaves confirmed the Stomata closure inhibition under moderate drought to maintain water transportation and a relatively high rate of carbon dioxide assimilation^15^. Molecular mechanisms covering the differential expression of the genes in *P. euphratica* under different stress levels have been described previously^16,17^. Different genes have been reported with altered transcript abundance including genes for the small HSP, HSP70 and HSP90 heat shock protein families, as well as members of the transcription factor families bZIP, AP2/EREPB, NF-Y, NAC, MYB and Homeobox and WRKY^14^. The reference genes, RPL17, HIS, EF1𝛼, TUB GII𝛼 and PeHAB1, from one-year-old seedling leaves, were found to be altered by various abiotic stresses. Genome-scale transcriptome analysis provides an extensive catalogue of the genes expressed in *P. euphratica* under different stress conditions i.e. PeuHsf gene expression was significantly induced by drought, salt and heat stresses^18^. The *Populus* special miRNA-target interactions^19^ might be involved in some biological process-related water deficit stress tolerance.

The *P. euphratica* have developed phreatophyte roots which deeply penetrate into the soil for moisture availability. Roots respond to a variety of below and above ground signals that modulate root system architecture^20,21^ by showing tropic growth responses. The root response is mainly governed by environmental stimuli^22,23^ as well as differential gene expression patterns and hormonal regulation^24^. Hypothetically, roots respond to and exploit the water gradients in the soil by the positive hydrotropism and grow towards increasing moisture, which explains the drought avoidance and precise exploitation of water patches^25–27^. The gene expression pattern changes with varying soil moisture and groundwater table^28^, showing a sensitive hydrotropic response. The fundamental and significant advances of the mechanism of hydrotropism and its interaction with gravitropism, at the cellular, molecular and genetic level, are not fully understood yet, as a little has been reported about the genetic players to explain how hydrotropism operates in the real world at molecular levels. Recent reports showed that MIZ1, MIZ2, AHR1 and NHR1 seem to play important roles in hydrotropism^27,29^. Similarly, the Ps-EXGT1 (XET) isolated from the roots of an agravitropic pea mutant, ageotropum, strongly expressed in hydrotropically responding roots^30^.

The *PsEXT* belongs to the large multi-gene family xyloglucan endotrans-glucosylases/hydrolases (XTHs)^31^, which mainly regulates cell wall strength and extensibility^32,33^. *MtXET*, *PsXET* homologous gene, from *Medicago truncatula* expressed significantly in root and showed the positive hydrotropic response, thereby helping in drought resistance^34^. Multigene families of XTHs have been reported in different plant species, including rice^35,36^, Arabidopsis^37^, wheat^38,39^, tomato^40^ and poplar trees species^41^. Their expression is tissue specific and is regulated by hormonal and environmental factors^36,42,43^, and play vital roles in various differentiation and growth processes, including: fruit ripening^40,44^, flower opening^45^, petal abscission^46^, vein differentiation^47^, wood formation^48^, hypocotyl growth^49^ and primary root elongation^50^.

*P. euphratica* developed some plasticity to adapt to the gradual environmental gradients such as the development of heteromorphic leaves and well-developed roots system. We investigated the physiological and molecular mechanisms enabling this species to cope with severe stress caused by drought. The microstructures of heteromorphic leaves were studied inside and outside greenhouse condition. The deep penetration of well-developed root system was studied in its natural habitat “Gobi desert” as well as in control condition. The root gene expression profiles of root system subjected to drought stress was monitored. The *XET* gene activated as hydrotropic responsive gene was cloned in accordance with DEGs information and its functions for root hydrotropism and drought resistance were confirmed in T2 transgenic tobacco.

## Materials and Methods

### Plant material and growth conditions

The drought tolerance characteristics, of *P. euphratica*, were investigated in National Natural Reserve of *Populus euphratica* Forest in Ejinaqi County, Inner Mongolia, China, in early spring. *P. euphratica* seeds and 2-year old seedlings were provided by the administration of the National Natural Reserve. Seedlings were planted in a container with 25 cm high and 12 cm diameter containing cleaned river sand, placed in a greenhouse with temperature/humidity (25 ± 5) °C/(60 ± 5%) in September, 2015. The containers were watered according to evaporation demand and supplemented with 1/2 MS medium solution once per week. After fully acclimatization to the new environment, part of the potted plants was kept out door in the natural condition, and rest of them were maintained in greenhouse.

### Leaf sampling and preparation for electron microscope scanning (EMS)

**Figure 1:**
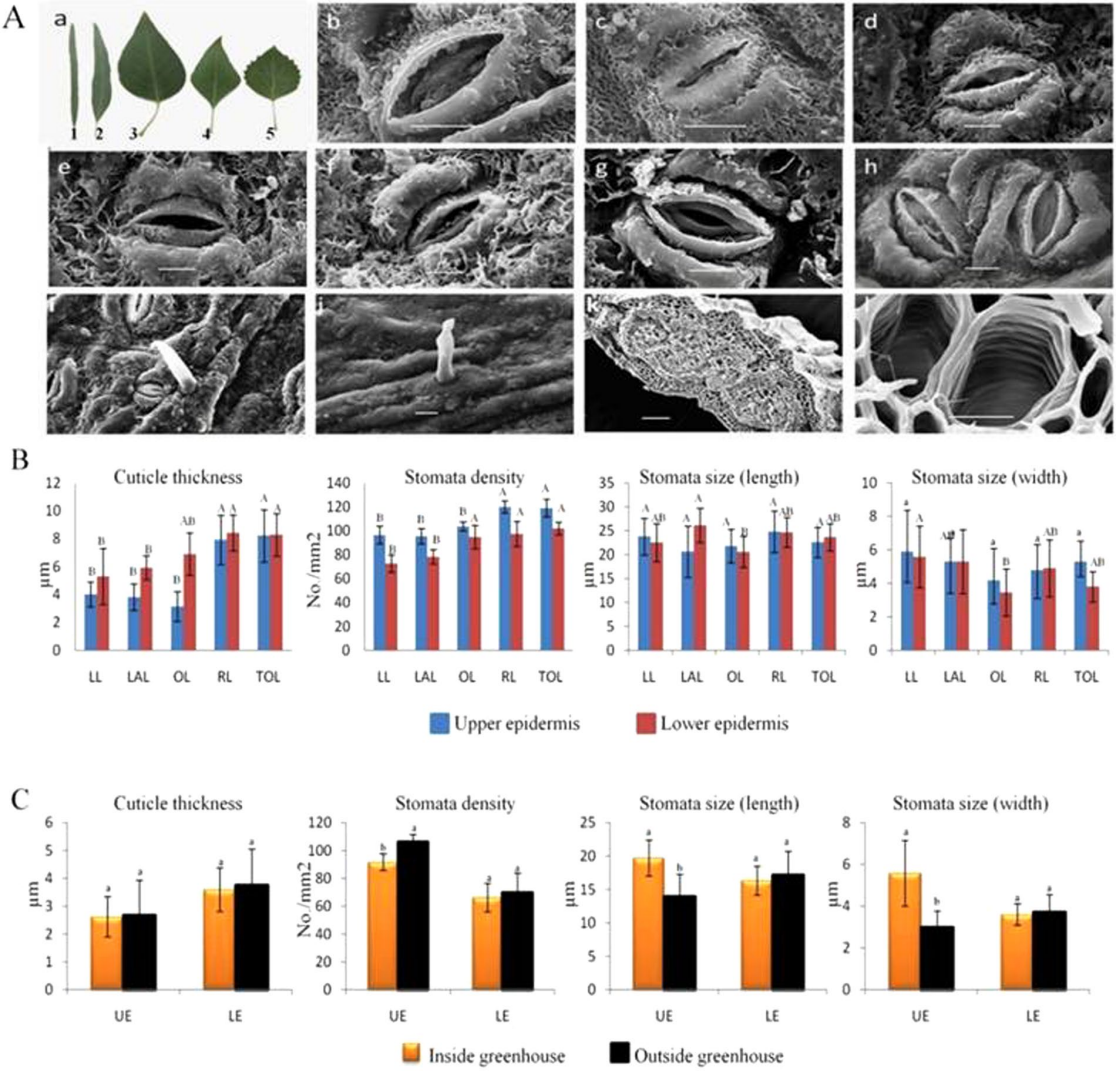
Scanning leaves with Electron Microscopic (SEM) in Populus euphratica. (**A)** (a) Adult plant five different heteromorphic leaves of 10-years old trees 1: Linear leaf (LL) 2: lanceolate leaf (LAL) 3: ovate leaf (OL) 4: rhombic leaf (RL) 5: toothed-ovate leaf (TOL). (b & c) LL stomata inside and outside greenhouse. (d & e) LAL upper and lower epidermis stomata outside greenhouse. (f & g) OL upper and lower epidermis stomata outside of greenhouse. (h) RL upper epidermis double stomata apparatuses (bar = 10 µm); (i & j) LL and RL upper epidermis trichome. (k) RL transection of principal vein of (bar = 100 µm) (l) LAL transection of spiral vessel in principal vein (bar = 10 µm). **(B)** Cuticle thickness, stomata density and stomata size (length and width) of upper and lower epidermis of P. euphratica. **(C)** Cuticle thickness, stomata density and stomata size (length and width) of linear leaf of 3-year-old P. euphratica inside and outside of greenhouse condition.

Five types of heterophylly were collected for EMS (Fig. 1A). Liner Leaves were sampled from 3-year-old plant grew inside and outside of the greenhouse conditions, other types of heterophylly were taken from over 10 years old tree nursery of Beijing Forestry University. The plant tissues were prepared, scanned and imaged with KYKY-2800 EMS. Each specimen was measured 5 times and their average value was taken. The data was processed with DPS 3.01 Data processing system.

### P. euphratica root architecture pattern in response to moisture level

**Figure 2:**
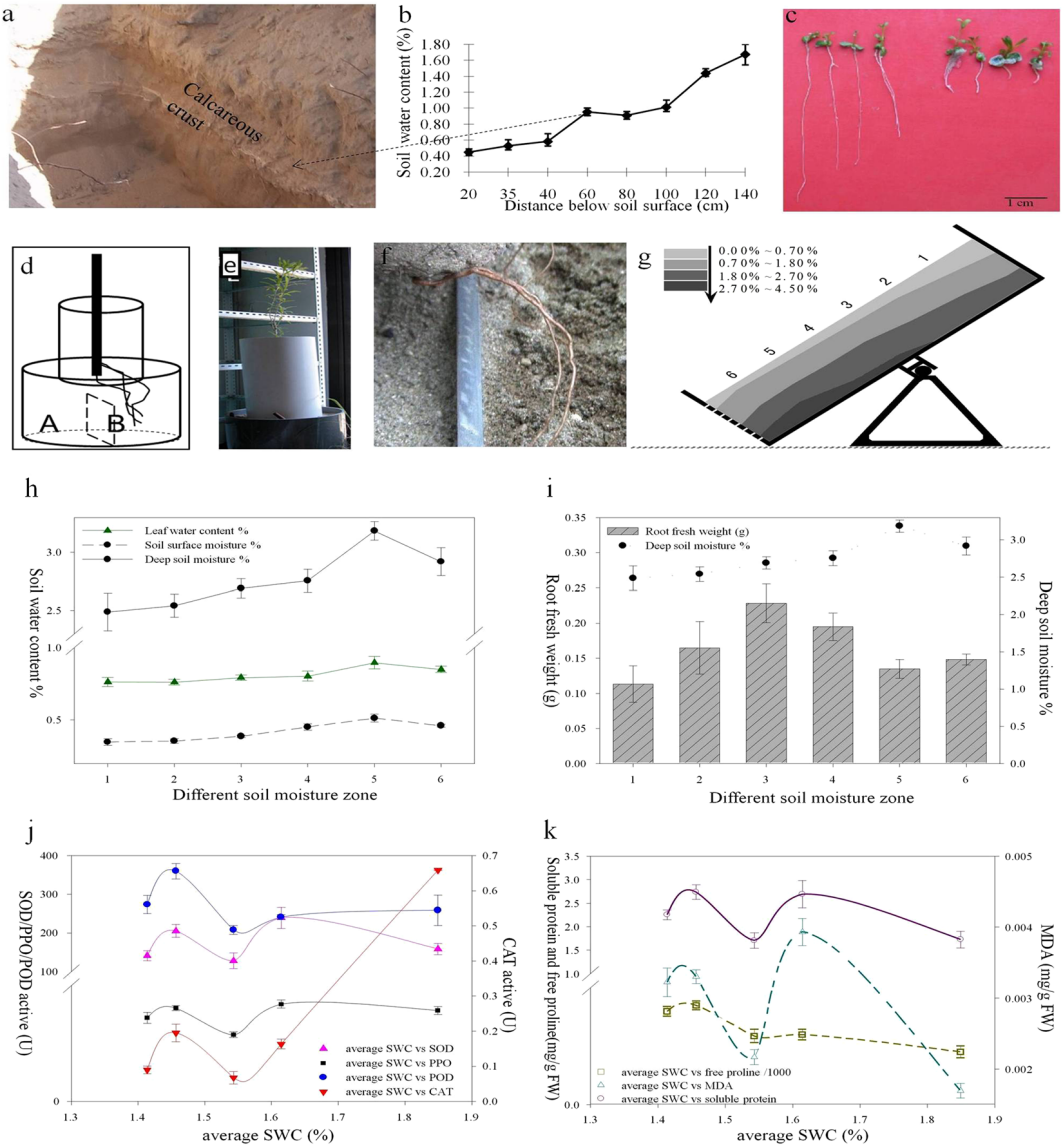
Root growth in natural habitat Badain Jaran Desert and in control (lab and greenhouse) condition and physiological parameters at moisture gradients. (**a**) Root section penetrating the hard calcareous crust at natural habitat. (**b**) Soil water contents of root zone at Badain Jaran Desert. (**c**) Root length of 30d old P. euphratica root seedling on MS media at moisture gradients. (**d**,**e**,**f**) P. euphratica root hydrotropism induction pot. (**g**) Specially designed horizontal cultivation equipment with 6 gradient moisture layer. Only the box left side had hole (Ø5mm × 8 rows × 8 columns). Down arrow followed by # % showing SWC difference in gradient soil drying. (**h**) Distribution of soil water (upper and deep soil water contents) and relative leaf water percentage in response to gradual soil drying. (**i**) Root fresh weight at soil moisture zone. (**j**) P. euphratica roots activity of antioxidant enzymes and the soil moisture profile. (SWC: soil water content; SOD: superoxide dismutase; POD: superoxide dismutase; CAT: peroxidase; PPO: polyphenol oxidase). (**k**) P. euphratica roots proline, MDA and soluble protein content.

The *P. euphratica* root response to soil moisture was investigated in field (Fig. 2a) and lab, respectively. A field survey was conducted in *P. euphratica* forest National Natural Reserve in Badain Jaran Desert, Egina Qi in Inner Mongolia of China (geographic coordinates: 41°56′57.93″N; 101°04′24.04″E). The soil profile of 10-year old plant was monitored and relative soil water contents and root pattern was recorded for every 20 cm soil layer.

In the lab, the seeds were surface sterilized and sewed on autoclaved solidified agar gel medium. Before allowing the media to solidify the medium container was kept slanted to achieve the varying levels of agar deposition within the same containers to achieve different level of water content. The *P. euphratica* aseptic seeds were cultured and germinated on the plain and slanted solidified medium. In greenhouse, a simple device was designed (Fig. 2d) to study the root growth pattern. The device consists of two cylindrical pots; the smaller one was hollow and placed on the main pot. The main pot had divided into two sub-pots A and B. Both sub-pots were filled with cleaned river sand, having embedded water pipe in side B for watering. Aseptic seedlings of *Populus* grown on MS media were transferred to plastic bags. After acclimatization in control condition, the plastic bags were removed and the plant was put on upper cylinder pipe and placed on the centre of the main pot (Fig. 2e).

### Root hydrotropism induction with reduced geotropism effects

A special chamber (length 180 cm × width 150 cm × height 45 cm) was designed to provide the natural plant growth and to exclude the influence of gravity^51^, having small pores (Ø5mm × 8 rows × 8 columns) along its width to maintain the natural flow of water. By rotating the designed apparatus by 60^o^ angle produced different moisture gradients (Fig. 2g) and reduced the interference of gravity. Chamber was filled with cleaned river sand and 3-year old *Populus* seedlings were grown in 6 lines (line space 30 cm) having 12 plants per line (row space 15 cm). The plant container was watered according to evaporation demand and supplemented with 1/2 MS medium solution once per week. After the growth of seedling to 15–20 cm, the growth chamber was tilted to obtain different moisture levels by withholding the water till the apex 1^st^ leaf wilting appeared in the chamber upper part (line 6). Soil water content (SWC) gradients and leaf water content were recorded with Psypro for all lines except line 6 to minimize false positive results. The fresh root morphology and fresh weight were recorded. The root samples were wrapped in foil and frozen immediately in liquid nitrogen prior to store at −80 °C refrigerator for microarray analysis.

### Leaf physiological index in response to soil different moisture levels

Leaves samples from *P. euphratica* plants grown in water deficit conditions were collected and physiological parameters proline content^52^, soluble sugars^53^, MDA^54^, polyphenol oxidase (PPO)^55^, plant peroxidase (POD)^56^, superoxide dismutase (SOD)^57^, hydrogen peroxide enzyme (CAT)^58^ and malondialdehyde (MDA)^59^ were analyzed.

### Microarrays analysis of root transcriptome in gradient soil drying

The microarray experiment was completed by Shanghai Biochip Company (SBC), China accredited by China National Accreditation Service for Conformity Assessment (CNAS). RNA was extracted from root sample and purified using the QIAGEN RNAeasy mini kit. The experimental lines (line 1–4 plant roots subjected to different drought stress) RNA was labeled with Cy3-dUTP and was henceforth described as regime 1, 2, 3 and 4, respectively. Control group (the 5^th^ line plant roots with sufficient moisture) RNA was labeled with Cy5-dUTP fluorescence and was henceforth described as regime 0. Further hybridization procedure was followed according to the chip manufacturer instructions (Shanghai Biochip Co., Ltd.).

Microarrays were scanned using an Axon microarray scanner (Angilent technology) and analyzed by split-tif Imagene software. Background fluorescence was subtracted from the value of each spot on the array. Normalization of the signal intensities was carried out according to Deyholos *et al*.^60^. Transcript regulation has been expressed as the ratio of intensities between stress and control root samples. Changes in signal intensity between stress and control experiments exceeding a >2.0-fold difference in repeat experiments were considered significant. GO, COG terms and KEGG pathways enrichment analysis was used for functional categorization of unigenes.

### RT-qPCR validation of microarray unigenes of P. euphratica roots in response to soil drying

Total root RNA was isolated from all lines (1–5) treated with RNase-free DNase (Chang *et al*., 2012). The root RNA samples were reverse-transcribed with a cDNA Synthesis Kit (CWBIO Inc., Beijing, China). cDNA products were used for SYBR Green-based RT-qPCR analysis, each sample was run in triplicate. The RT-qPCR running conditions were: 95 °C for 10 min, followed by 40 cycles of 95 °C for 15 s, 52 °C for 20 s, and 72 °C for 30 s, with a final step of 72 °C for 10 min. Using the roots of line 5 group plants as control, the expression levels of differential expression genes (DEGs) were calculated using the 2^−∆∆Ct^ method^61^.

### Functional analysis of PeXET gene and promoter in root response to soil drying

According to the sequenced results of the microarray analysis and that of *Ps-EXGT1* (GenBank Acc. AB015428)^30^, a pair of primers (PeXET-722–1 and PeXET-999–3, Table S1) were designed to obtain the ORF of *PeXET*. The PCR running conditions were: 94 °C for 3 min, followed by 35 cycles of 94 °C for 40 s, 55 °C for 30 s, and 72 °C for 1 min with a final step of 72 °C for 3 min. After electrophoresis, the PCR product was extracted from the agarose gel using Agarose Gel Extraction Kin (TianGen, Beijing) and inserted into T-vector for sequencing (Sangon Biological Engineering Co., LTD). 3′ RACE and 5′ RACE were used to obtain the full-length cDNA of *PeXET* according to Molecular Cloning Manual: A Laboratory Manual (Chapter 8), the sequences of primers shown in Table S1.

The *PeXET* gene function was analyzed in transgenic tobacco. pBin438-*PeXET* and pBin438-RNAi-*PeXET* vectors were constructed by picking primer sequence from *PeXET* gene and *GUS* sequences (Table S1 and Figure S3). GV3101/ pBin438-*GUS*, pBin438-*PeXET* and pBin438-RNAi-*PeXET* were delivered into tobacco (*Nicotiana tabacum*) through agrobacterium mediated transformation^62^, respectively. Marker resistance shoots were tested by PCR and T2 generation plants were subjected to RT-PCR analysis. The RT-PCR products were sequenced and analyzed by DNAMAN sequence alignment software.

Transgenic tobacco seeds were surface sterilized (8 seeds of each transgenic pBin438-*PeXET*, pBin438-RNAi-*PeXET*, and pBin438 plants), and germinated on MS media in Petri dishes. The germinated plants were investigated for 14–20 days for the seedling growth and development. The plants from three sort T2 generations were transplanted into a box (67 cm × 47 cm × 18 cm) and care was taken to gain the height of 15 cm. The plants were uprooted and root-shoot morphology was recorded. For drought tolerance test the plants grown in separate boxes were subjected to water withholding treatment. Soil water contents, the plant apex leaf wilting and survival rates were recorded.

The *PeXET* gene promoter (*pPeXET*) was cloned to insight its gene driven activity^63^. The promoter region was double digested to elucidate their function with restriction enzymes HindIII and NcoI, created sub-sequences i.e. 1831bp, 1425 bp, 993 bp, 717 bp and 588 bp *PeXET* 5′-flanking sequences. In pCAMBIA expression vector, CaMV35S promoter was substituted with the *pPeXET* (1831 bp) and 5′ end deleted portion of *pPeXET* (588 bp), respectively. The expression vector pCAMBIA-*GUS*, pCAMBIA-*pPeXET1831*-*GUS* and pCAMBIA-*pPeXET588*-*GUS* were transformed into GV3101 via freeze-thaw method^64^. GV3101/ pPeXET1831-GUS, pPeXET588-GUS and pCAMBIA-GUS vector (control) were transformed into tobacco. Hygromycin resistance tobacco plants were confirmed by PCR using *GUS* upstream primer 5′-AGCGTTGAACTGCGTGAT-3′ and downstream primer 5′-GTTCTTTCGGCTTGTTGC-3′. Histochemical staining and fluorescence quantitative analysis were performed to identifying the promoter activity derived *GUS* expression in the transgenic tobacco plant systems^65^.

### Data analysis

Data were subjected to analysis of variance (ANOVA) and differences between means were evaluated with Student′s *t*-test. The differences were considered statistically significant at *P* < 0.01 and *P* < 0.05. Statistix v. 8.1 (Analytical Software, 2005) package was used for this purpose.

## Results

### Heterophylly characteristics of Populus euphratica in response to environmental conditions

The adult plant leaves of *P. euphratica* are heteromorphic. The leaf showed the disciplinary change from lanceolate leaves to dentate broad-ovate leaves. At least five different leaf shapes were found in a tree of *P. euphratica*, i.e. Linear leaf (LL), lanceolate leaf (LAL), ovate leaves (OL), rhombic leaf (RL) and toothed-ovate leaf (TOL) (Fig. 1A). Attending to the different cutin structure, *P. euphratica* leaf cutins have been grouped into two general morphological types. (i) Compact layer, the black compact structure covers the leaf surface continuously or disconnectedly. (ii) Loose layer, the white color covers the leaf surface in a crisscross stagger network and honeycomb-like structures. Cutical thickness was fond higher in lower epidermis (LE) as compared to the upper epidermis (UE). The cutin coverage ratio increased in the order: LL<LAL<OL<RL<TOL (Fig. 1B). Cutin layers were found higher in old trees (10 year old) than younger plants (3 year old), while no significant difference was observed for the leaves growing inside and outside of greenhouse (Fig. 1C).

Stomata of the *P. euphratica* tree leaves are rectangular round and distribute on both UE and LE, raise or sink parasitically in the cuticle layers (Fig. 1A). Outer stomata ledge swells up outwards and forms the front chamber and communicates with the environment. Stomata density on LE and UE were found same for all the heteromorphic leaves except for OL (Fig. 1B). Whether UE or LE, the stomata density in the five heterophylly from lower to higher is in the order: LL <LAL<OL<RL<TOL, respectively. Stomata size on upper and lower epidermis showed significant difference growing inside and outside of greenhouse (Fig. 1C).

The UE stomata size was found longer than LE except for LAL. The maximum length was 5.4 µm in the LAL and the minimum one is 0.22 µm in RL. The stomata width from UE and LE were similar in length, the maximum width was 1.44 µm in TOL and the minimum was 0.04 µm in LAL. While the UE stomata sizes showed no significant difference among the five heterophylly (*P* < 0.05). In greenhouse condition, the stomata length and width of UE in leaves of 3-year- old plants of *P. euphratica* were significantly longer and wider than open field plants, while no significant difference found in the case of LE (Fig. 1C).

Leaf trichomes occurred on both epidermal side of RL and on the UE of LL in the 10-years old trees, other heterophylly growing inside or outside greenhouse were without trichomes. The maximum trichome length was recorded as 51.58 µm and base diameter of 12.67 µm.

Three vascular bundles, one smaller and two bigger, were found in RL principal vein, smaller one was located between two bigger ones. However, only one vascular bundle appeared in other heterophylly from 10 years-old plants and the 3-year-old *P. euphratica* plants growing both inside and outside greenhouse. The vascular bundle sheath was composed of 2–5 layers of stereid cells arranged closely. 4–8 layers of parenchyma and sclerenchyma cells developed between the vascular bundle sheathes and epidermis, meanwhile, the parenchyma cells or the sclerenchyma cells were different in sizes. Vessels of vascular bundles in the principal vein were mainly spiral vessel with 11.04 ± 6.31 µm in diameter.

### Root growth in response to Badan Jaran Desert soil moisture in P. euphratica

Root distribution, relative soil water content of the soil profile and other features were studied as shown in Fig. 2a and b. In the Badain Jaran desert, soil water content increase gradually from up to down, *Populus* root were found to penetrate the hard calcareous crust (50–60 cm below from the soil surface) as the moisture level found double below the crust (salt deposits).

In the lab, the seedlings were grown on water potential gradient media with low water content at the top as compared to the lower part. Accordingly, 30 days old seedling roots were significantly longer at the top zone as compared to the root length at low zone (Fig. 2c). In green house, *Populus* seedlings were tested in specially designed apparatus as shown in Fig. 2d and after five months of regular irrigation to one side, the plant roots were found bent deeply in the soil chamber with high moisture gradients (Fig. 2e,f). Results showed the strong hydrotropic response of *Populus* roots toward moisture.

### Physiological responses in leaves to soil water stress in the water-withholding regimes

The *P. euphratica* plants showed distinct physiological features when subjected to different moisture gradients. At different soil moistures zones (lines 1–6) in the same cabinet (Fig. 2g), *P. euphratica* roots were differed in the terms of growth and fresh weight. The highest fresh weight of roots was achieved in line 3 (average SWC 2.71%), the soil moisture higher or lower than this, such as line 4 (average 2.79%) and top line (SWC less than 2.50%), reduced the fresh root growth (Fig. 2i).

Physiological parameters of leaves subjected to water withholding are shown in Fig. 2h–k. Several antioxidant enzymes, SOD, PPO and POD showed a relatively stable expression at earlier drought stress but further reduction in soil water content caused the decline activity. When the soil moisture levels reduced to a certain level the enzymatic activity showed a transition stage and gain high activity. CAT activity showed a rapid decline as SWC fall down but further reduction of soil water moisture caused the up-regulation. In contrast, SOD showed a slight increase activity to deficit water condition but after that further reduction in moisture level caused decline and then gain high activity. Soluble protein, MDA and proline concentration showed rising trend against to reduced SWC but further decline trend followed the reduction in SWC, while a sharp decline trend was in MDA content as shown in Fig. 2k.

### Microarrays (chip) analysis of gene expression profile in roots of P. euphratica in response to soil drying

Data analysis of the four regimes (i.e. the individual drought treated regimes compared to regime 0) revealed that the DEGs showed differentially expressed transcripts profiles in response to the moisture gradients. A total of 1394 transcripts were significantly up and down-regulated in all four regimes compared to regime 0. Regime 2 and regime 3 showed the highest number of specific, differentially expressed transcripts (124 and 128 up-regulated, 312 and 249 down regulated, respectively), in comparison to regime 1 (160 up and 166 down regulated) and regime 4 (122 up and 136 down-regulated genes).

**Figure 3:**
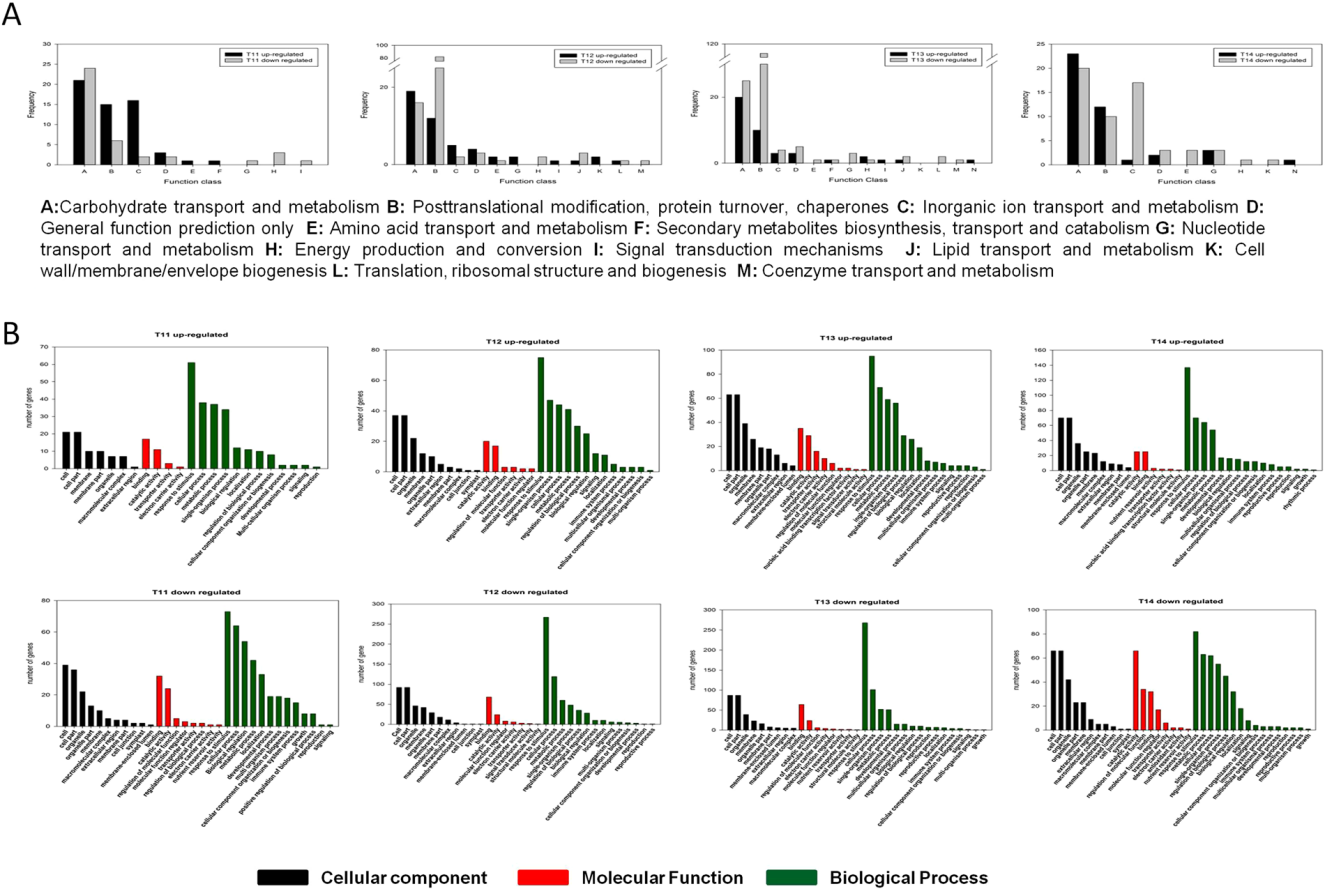
Transcriptome analysis of P. eupheritica roots at moisture gradients. (**A**) COG classification of differentially expressed genes (DEGs). Total of 531 DEGs were classified into different groups; (**B**) Functional classifications of GO terms of all DEGs. Total unigenes were classified into three main groups namely: cellular component, molecular function and biological process.

The transcriptional information was obtained by BLAST software and the unigenes were assigned to NR, Swiss-Prot, GO, COG, and KEGG database (Table 1). A total of 835 unigenes were identified, in which531 unigenes were analyzed by COG (Fig. 3A), 515 unigenes were enriched in GO database (Fig. 3B) and 287 unigenes were mapped to KEGG pathways (Table S4).

**Table 1:** Summary of unigene annotations.

Annotation database	Annotated number	300 <= length < 1000 (bp)	length >= 1000 (bp)
COG	531	50	481
GO	515	86	427
KEGG	287	32	255
Swiss-Prot	724	95	627
NR	835	112	719
All annotated	835	112	719

GO: Gene Ontology; COG: Cluster of Orthologous Groups of proteins; KEGG: Kyoto Encyclopedia of Genes and Genomes.

The functions of the differentially expressed genes (DEGs) were searched by GO database for plotting annotation results. A total of 515 DEGs were categorized into three main categories (cellular component, molecular function and biological process) and 42 functional groups (Fig. 3B). Annotations of DEGs assigned into the GO database showed different moisture levels changed the expressions of the transcripts in stressed roots (Table S3). Overall results of GO database annotation showed that 198 DEGs involved in the catalytic reaction pathways (catalytic activity), 251 DEGs belong to protein binding ways (Binding), 331 DEGs involved in metabolic pathways of exogenous stimuli (response to stimulus), 138 DEGs involved in the biological regulatory pathways (biological regulation). The highest numbers of transcript changes were observed for regime 3 followed by regime 4. In regime 3, highest number of transcripts 95 and 69 involved in “response to stimulus” and “cellular process” were up-regulated.

Based on COG analysis, total of 531 DEGs were classified into 26 groups (Fig. 3A). According to COG annotation, 225 DEGs involved in post-translational modifications, protein folding, chaperones metabolic pathway, 173 DEGs involved in carbohydrate transport and metabolism pathways, 40 DEGs involved in lipid transport and metabolism, 28 DEGs involved in the inorganic ion transport and metabolic pathways.

### Expression profiles of stress-inducible transcriptomes in response to soil drying

In microarray data, unigenes belonged to the same family were in different expression patterns. Unigene for inositol-3-phosphate synthase was up-regulated 6.557 fold in regime 3, while 2.885 fold increased regime 4. The same unigene was down-regulated in regime 2 as its expression was 0.218 (Table S2). A total of 24 unigenes for inositol-3-phosphate synthase were up and down-regulated in the roots sample subjected to different soil moisture levels. Phosphatase 2C as a regulator of ABA signaling was down-regulated in regime 1 by 0.373 fold expression and up-regulated in regime 3 by 2.052 fold. Stress responsive signaling molecule “mitogen-activated protein kinase kinase 2 (MAPKK)” in regime 3 *P. euphratica* plant roots increased 5.412 folds, while in regime 2 it was down regulated 0.299. NAC TF was up regulated 2.282 and 3.036 times in regime 4 and regime 3, respectively.

Unigenes for histidine-containing phosphotransfer proteins (HPts) which act as cytokinin signaling receptor was up-regulated by 2.532 folds only in regime 3. Similarly, homeobox-leucine zipper protein was up-regulated by 2.116 folds and 3.193 folds in regime 4 and regime 3, respectively. In the *Populus* root regime 1 and regime 2, the auxin-responsive protein was up-regulated by 2.5 fold.

Unigenes for stress responsive transcription factor was up-regulated in regime 3 and regime 4 by 3.036 and 2.282 folds, respectively. High water content levels up-regulated the expression of SKP1-like protein while below water content levels up-regulated the expression trend of BON1-associated protein 2 which act as cell death regulator. Details of up and down regulated unigenes are given in Table S3.

### The expression pattern of XET gene involved in root hydrotropic growth

In microarray data, several *XET* transcripts were found to be up-regulated at different moisture levels. Six transcripts of *XET* were up-regulated specifically according to the level of drought, such as four transcripts were up-regulated (2.556, 2.854, 3.199 and 3.471) in regime 4. Similarly, three transcripts of *XET* were up and down regulated (2.445, 0.429 and 0.128) in regime 3. For *XET* transcripts in regime 2, same trend was observed with up and down regulated of 3 transcripts (2.355, 0.446 and 0.374). The *XET* activities remained highest for regime 3 and were selected for the further analysis.

### Validated the DEGs from microarray analyses by RT-qPCR analysis

For validating the data from microarray, 26 DEGs were randomly selected for RT-qPCR analysis at different moisture levels. The primers of selected genes are listed in Table S1. *PeActin* was used as reference gene for data normalization according to *Hu et al*. 2015^66^. The RT-qPCR results showed a strong correlation with the RNA-seq-generated data with few exceptions. Only two genes encoding protein phosphatase 2 C and suspected new gene displayed a higher expression in the microarray data as compared to RT-qPCR analysis.

### Functional analysis of PeXET promoter and its spatial expression profile

Analysis of the promoter sequence (1831 bp) showed different cis-elements mainly involved in growth regulation, growth and environment responsive element. Most of growth response cis-elements and growth regulator cis- elements fall in 588 bp sub-sequence of the *pPeXET* promoter region (Table 2).

**Table 2:** *PeXET* promoter cis-regulating elements functions prediction analysis by PlantCARE software.

Element name	Function	No.	Sequence
**Growth regulator cis-element**			
TGA-element	Auxin responsive element	1	AACGAC
P-BOX	Gibberellin-responsive element	2	CCTTTTG
			CCTTTTG
ERE*	Ethylene responsive element	1	TTTGAAAT
TCA-element*	Salicylic acid response element	1	CATTCTTCTC
TGACG motif*	MeJa responsive element	1	TGACG
CGTCA motif*	MeJa responsive element	1	CGTCA
**Growth response cis-element**			
CCGTCC BOX*	Plant meristem special regulating element	1	CCGTCC
CAT-BOX*	Meristem expression inducing element	1	GCCACT
Skn-I-motif*	Endosperm specific expression element	1	GTCAT
TATCCAT/C- motif	Associated with G-BOX motif involved in sugar repression responsiveness	1	TATCCAT
EIRE	Elicitor responsive element	1	TTCGACC
AC-II*	Lignin transporting responsive element	1	CCATCAACCCCC
AS-2-box*	Shoot specific expression inducing element	1	GATAATGATG
circadian*	Biological clock controlling element	1	GATATCTTA
**Environment response cis-element**			
ARE*	Anaerobic responsive element	1	AAACCA
GC motif*	Strengthen the anaerobic reaction element	1	CCCCCG
MBS	Drought inducing responsive element	2	CAGTTG
			ACCG
BOX E	Lack of water responsive element	1	ATGGGT
GAG motif*	Light responsive element	1	AGAGATG
TCT motif	Light responsive element	2	TAAGA
			GTAAGA
SPI	Light responsive element	2	CCACCCATGC
			GGGGGGCACC
G-BOX	Light responsive element	3	ACAAGTGGT
			CACGTT
			ACATGG
BOX 4	Light responsive element	1	ATTAAT
DRE	Abiotic sress response element	1	RCCGAC
BOX I	Light responsive element	1	CGGGGG
WUN motif*	Mechanical damaging responsive element	1	AGGAAATTT
TC RICH repeats	Defense and stress responsive element	1	ATTTTTTTCA

Element name followed by *showed that this element present in 588 bp fragment.

**Figure 4:**
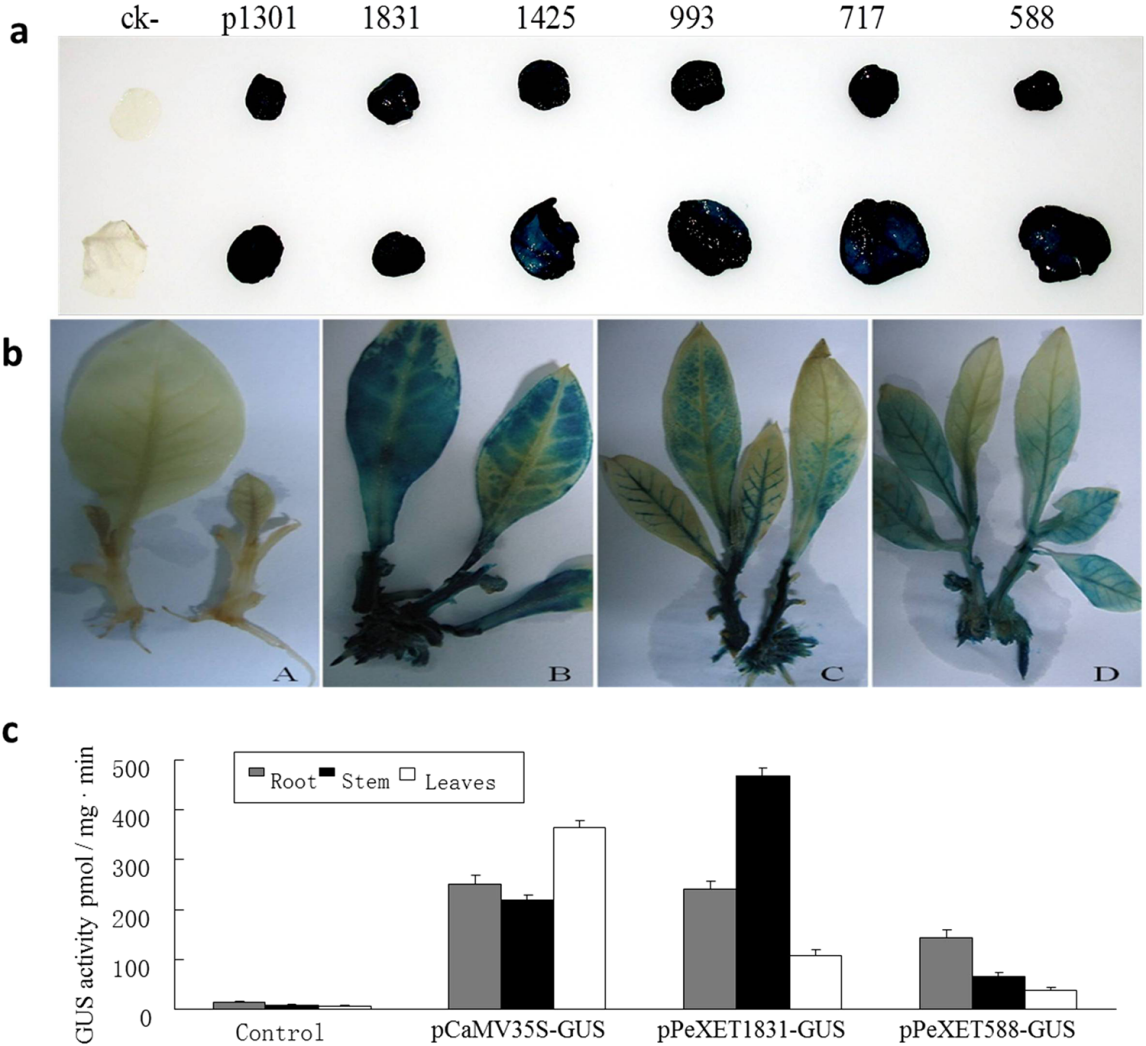
PeXET promoter driven activity analysis (**a**) promoter driven GUS gene expression. (**b**) GUS tobacco histochemical staining. (**c**) GUS expression activities of transgenic tobacco at different. organs.

1831 bp and 588 bp fragments *pPeXET* were analyzed for their *GUS* driven expression activity in transgenic tobacco. The Hygromycin resistance transgenic tobacco shoots of *pPeXET1831*-*GUS*, *pPeXET588*-*GUS* and control *pCaMV35S*-*GUS* were confirmed by PCR and sequence analysis^63^. The GUS gene expression in the transgenic tobaccos is given in Fig. 4, GUS staining showed blue colors in roots, stems and leaves in the transgenic tobacco but the color intensity was different from dark to light (Fig. 4b). Quantification of Fluorescence of 4-MU (Fig. 4c) showed that the *GUS* expressions activities vary for the different used promoters and transgenic plant organs (root, stem and leaves). *pPeXET1831* driven *GUS* expression levels was similar in roots, highest in stems while diminished in leaves as compared to CaMV35S. Lowest *GUS* expression level was recorded for *pPeXET588* fragment which showed a decreasing trend of expression in roots stems and leaves, respectively.

### PeXET gene function in root growth and drought resistance in transgenic tobaccos

*PeXET* gene sequence was 2167 bp in length, included four exons and three introns (Figure S2) with length of 132 bp, 143 bp and 892 bp, respectively. The four exons were spliced together according to the rule of GT-AG as that of most plants. *PeXET* ORF sequence was cloned and submitted to GenBank (Accession No EF612703). The ORF sequence was 1224 bp, included the start codon (ATG) and stop codon (TAA), and encoded a 293-amino-acid residue with predicted molecular weight of 33.8 kDa. The protein sequence blast analysis subjected to NCBI TBLASTN homology alignment showed more than 90% similarities to the Populus XTH (more than 90%), and 83% to the trigged gene Ps-EXGT1.

**Figure 5:**
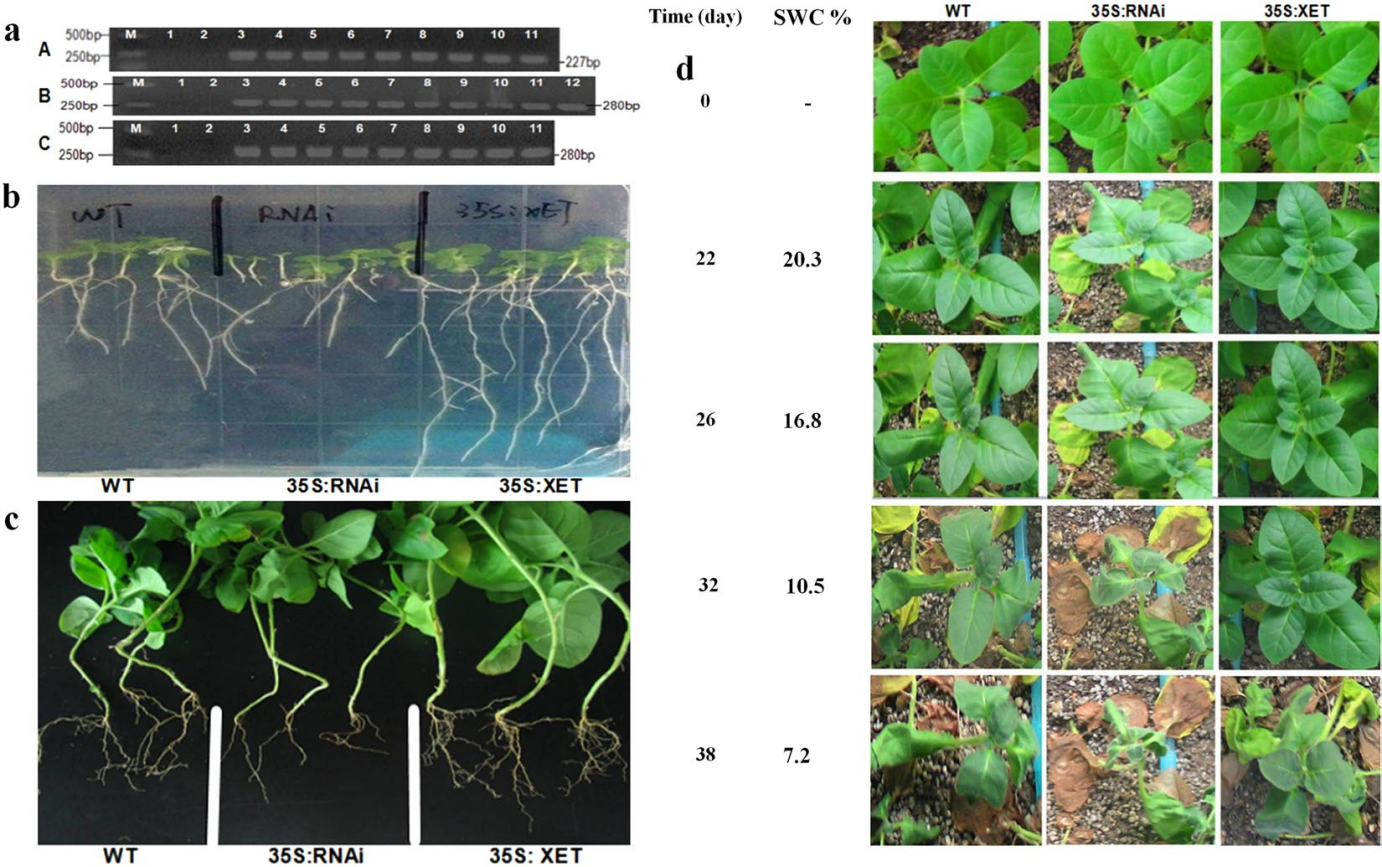
PeXET transgenic and RNAi tobacco plant root growth and drought tolerance. (**a**) T2 transgenic tobacco RT-PCR, A: M: Marker; 1: Vector control; 2: GV3101/ pBin438-35S-peXET; 3–11: transgenic pBin438- PeXET tobacco; B: M: Marker; 1: Vector control; 2: GV3101/ pBin438–35S-peXET; 3–12: pBin438-RNAi-PeXET tobacco; C: M: Marker; 1–2: Negative control; 3–11: transgenic pBin438 tobacco. (**b–c**) Tobacco roots of WT, RNAi and over-expressed PeXET gene plant on medium and pot soil, respectively. (**d**) T2 transgenic tobacco plant survival rate of WT, RNAi and over-expressed PeXET gene at different SWC.

The transgenic tobaccos were identified by PCR and T2 transgenic tobaccos were analyzed by RT-PCR (Fig. 5a). Total of nine vector controls, nine pBin438-*PeXET* transgenic tobacco plants and 10 pBin438-RNAi-*PeXET* transgenic plants were confirmed through RT-PCR and analyzed by DNAMAN software sequence alignment (data not shown).

Seedling roots architecture of WT, RNAi and *PeXET* over expressed transgenic tobaccos lines on *in-vitro* MS medium were shown in Fig. 5b. The longest root length, high fresh weight and rapid growth were recorded for pBin438-*PeXET* transgenic plants, while significantly inhibited root numbers and growth were recorded for pBin438-RNAi-*PeXET* plants. The upper parts of the WT, vector control and pBin438-*PeXET* transgenic plants showed no significant difference. The three transgenic lines grown in soil and cultured in greenhouse suggested statistically significant differences in root length, root fresh weight and lateral root numbers (Figure S4b and S4c). As compared to WT, pBin438-*PeXET* transgenic plant root number and length was high with more number of lateral roots. Short roots and few of lateral roots was recorded for pBin438-RNAi-*PeXET* plants, even main taproot was absent in the plants (Fig. 5c).

The *PeXET* T2 transgenic plants were subjected to drought stress by withholding water and soil drying naturally. RNAi transgenic T2 plants (pBin438-RNAi-*PeXET)* were found drought sensitive in response to the soil drying. Leaves of the plants wilted earliest (Fig. 5d) at the 22^nd^ day of withholding water with SWC 20.3%. WT tobacco plants appeared wilting at 26^th^ day of drought stress (SWC 16.8%), at this time stomata conductance was found higher in transgenic plants as compared to WT (Figure S4a). The pBin438-*PeXET* transgenic plants were found healthy at 10.5% SWC. The *PeXET* over-expressed plants did not appear wilting at apex first leaves until 38 days of drought stress (SWC 7.2%). The drought resistance tests suggested that *PeXET* gene increased the transgenic tobacco root growth and enhanced the plant drought tolerance.

## Discussion

In this study, the drought tolerant characteristics, the heteromorphic leaf and hydrotropically responding root system, of the *P. euphratica* were studied in soil water deficit conditions. The leaf covers, stomata architecture, physiological and biochemical changes, and characteristic gene expression of the spatial heterophylly from below Linear leaf (LL) to upper toothed-ovate leaf (TOL) showed the adaption pattern to the dramatic alteration in environmental air conditions. The extremely well-developed root system, characteristic microarray DEGs and individual *PeXET* gene expression driven by its root special *PeXET* promoter suggested that the plant root hydrotropism plays an essential role in adaption to soil moisture from the soil surface to deep groundwater.

The changes in the molecular sizes of heteromorphic leaves, especially stomata size and density and vascular bundles of the *P. euphratica* (Fig. 1), are considered to have a functional significance in response to fluctuating air condition. Morphological variation in leaf traits occurs from linear leaf (LL) to toothed-ovate leaf (TOL) according to the canopy position as the plant grows from young plant to big tree. The leaf cuticular thickness (Fig. 1B) was statistically classified into two groups (LL, LAL and LO; RL and TOL) on the upper epidermis and three groups (LL, LAL; LO; RL and TOL) on the lower ones. The epicuticular wax (hydrophobic layer) coverage increased from LL to TOL in the heteromorphic leaves (Fig. 1B). This cuticular protector is considered as a self-defense mechanism to reduce water loss through the trichomes and epidermis transpiration^67^ in water deficit condition.

Euphrates poplar showed difference in the stomata density, length and width on upper and lower epidermis as well as in the different heterophyll (Fig. 1B). Environmental conditions inside and outside greenhouse (mainly the humidity difference) affected the stomata densities of upper and lower epidermis and the stomata length and width of upper epidermis but not of the lower epidermis (Fig. 1B). Data suggested that the plant spatial air conditions control the stomata apparatus (open and close state) by affecting the stomata density, size length and width in upper and lower epidermis. This complex stomata apparatus and deep sunken stomata control the water balance by managing the transpiration rate according to the plant spatial air conditions^68^. The heteromorphic leaf of *P. euphratica* showed the close resemblance of xeromorphic characters which maintain normal growth and development even under undesired conditions^11^.

Mechanisms involved in the plant drought resistance were elucidated in physiological analysis at different moisture gradients. When the plant were subjected to soil drying, SOD, POD, PPO and CAT activity shows varying trends (Fig. 2j), offers improved protection against oxidative injury induced by drought. The fluctuating activities of MDA, free proline and soluble protein (Fig. 2k), enhance the plant drought stress resistance. The similar effects were studied in some model plants^69–71^. Antioxidant molecules and enzymes that are situated in various cell sections can scavenge ROS. Depends on the plant species, organ, developmental stage and the severity of the stress expression of the antioxidant capacity fluctuate. To keep the ROS concentration relatively low and acclimatized to water deficit condition is generally linked with improved activity of the antioxidant enzymes^72^. Beside this, ROS also utilized as secondary messengers in the signaling for the activation of defense responses. Tolerance to water stress circumstances is a complex feature accomplished by plants through coordinated action of biochemical, physiological and molecular adaptations. The bio-synthesis of osmotically-active substances, non-enzymatic and enzymatic antioxidants, contributes a key role in tolerance development against water deficit condition in Poplar^73^. Thus the varying expression trends of physiological parameter might be important for the *P. euphratica* to minimize the injury caused by drought stress and to fully activate the defense responses. Similar results for the same species were also reported by Song *et al*., 2014^74^.

The root system of *P. euphratica* was characterized with hydrotropism in response to natural growth condition (Fig. 2a), confirmed in lab (Fig. 2c) and greenhouse (Fig. 2f). At organ level, the dynamic responses to limited water availability result in an intricate pattern of roots within the soil, and this emerging characteristic finally specifies the extent of water accessibility in the soil. To date, in the field of plant breeding, rather static, idealized root phenotypes, namely ideotypes, have been targeted to optimize plant growth under a particular environment or stress condition. To understand the actual molecular mechanism of root phenotypes, tree physiologists focused on gene expression profile of plants across a wide range of environmental conditions^75,76^ to unhide the basic theme of root development of the woody plants in different environmental conditions.

**Figure 6:**
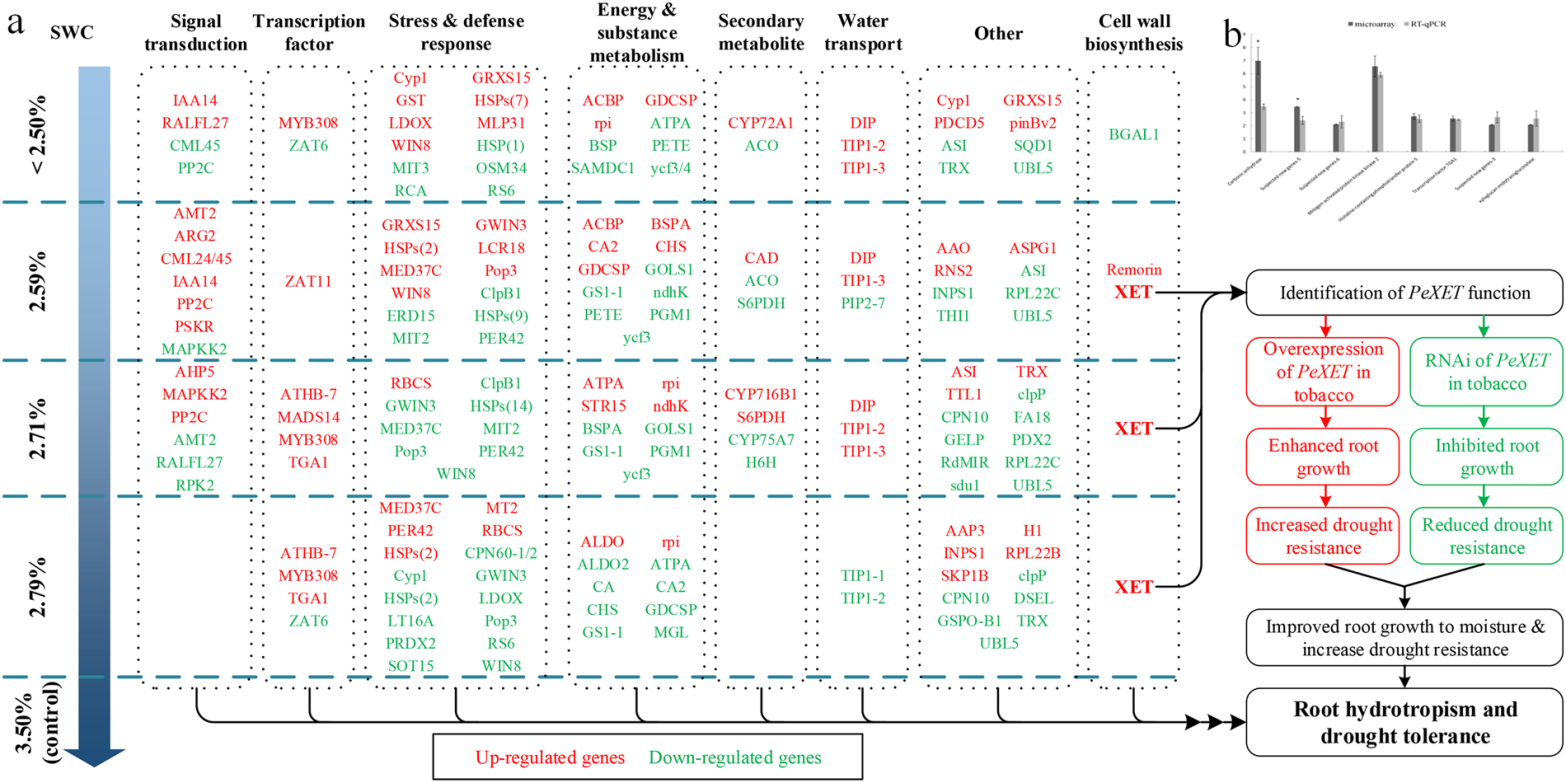
(**a**) A diagram of the DEGs pathways in response to soil moisture gradients (SWC 2.50, 2.59, 2.71, 2.79 and control 3.50%). (**b**) Validate Microarrays data by RT-qPCR. Expression level followed by *show the significant difference between Microarray and RT-PCR expression.

Microarray data showed that unigenes belonging to the same family have different expression patterns (Fig. 6a). Recent studies have reported that plant root elongation and lateral root growth dependent on the regulation of growth hormone^24^. Auxin biosynthesis, signaling and responding are required for lateral root formation, but the high concentration of auxin inhibit the lateral growth. In the popolus root of regime 1 and regime 2 the Auxin-responsive protein was up-regulated by 2.5 fold, indicated that the Populus roots experienced drought stress and retarded the root growth in the stress environment. Class phosphatidylinositol phosphate protein is an important component that promotes auxin signal and affecting cell membrane tightness^77^. Maximum number (24 unigenes) of phosphatidylinositol phosphate protein was up and down-regulated in the different moisture level roots sample, suggesting that the *P. euphratica* cell membrane is extremely sensitive to moisture gradients. Unigenes for inositol-3-phosphate synthase was up-regulated 6.557 fold in regime 3, while 2.885 fold increases was studied for regime 4. The Same unigene was down regulated in regime 2 as its expression was 0.218. In higher plants, myo-inositol becomes incorporated into myo-inositol phosphate (InsP), certain sphingolipid signaling molecules and phosphatidylinositol phosphate (PtdInsP), that participate in many biological processes, such as gene expression regulation^78^ and stress tolerance in particular^79^. Unigenes for histidine-containing phosphotransferproteins (AHP) which act as cytokinin signaling receptor was up-regulated by 2.532 fold only in regime 3 Populus root. AHP take part in cytokinine signal transduction in higher plants^80^ and play important roles in the development of several shoot and root organs through maintenance of cell proliferation activity^81^. Salicylic acid responsive protein (Transcription factor TGA1) was up regulated in regime 4 and regime 3, previous studies showed the direct involvement of TGA (salicylic acid responsive protein) in lateral root development. These results showed that hormonal regulation has the direct influence on root growth and their regulations alter with soil water gradients.

In addition to plant hormone pathways, several transcriptional factors and vital genes were found to be regulated by water availability. MAPKK in regime 3 *Populus* root showed 5.412 fold increases, while in regime 2 it was down regulated 0.299. Mitogen-activated protein kinase (MAPKs) cascades are signal transduction modules regulating various aspects of plant biology, including stress responses and root growth^82^. Lopez-Bucio *et al*.^83^, described the direct involvement of MAPKKs in root formation and architecture. NAC transcription factors, was up-regulated and members of the NAC gene family have been suggested to play important roles in the regulation of the transcriptional reprogramming associated with plant stress responses^84^. Different moisture level may accelerate cell wall stiffening possibly mediated by peroxidase. Data showed that 8 peroxidases transcripts were found to be differentially expressed with 7 down-regulated and 1 up-regulated in different root transcripts. The down-regulation of those peroxidases transcript may favor root elongation by the reduction of apoplastic H_2_O_2_, and the enhancement of oxygen radicals to break the cell wall polymers and as a result, accelerates cell wall loosening and therefore roots growth^85^. Unigene for Stress responsive transcription factor was up-regulated in regime 3 and regime 4 as a result the self-defense system of the plant became fully operative in optimum environmental condition. On the other hand, plants grown at drought stress condition, BON1-associated protein 2 was found up-regulated, which act as Cell death regulator. Results showed that each key gene plays an important role in root elongation under fluctuating moisture environment.

Transcriptome analyses of different moisture level indicate that the expression of genes related to cell wall stiffening and loosening is differentially expressed. Several XET transcripts were found to be up-regulated in different moisture levels (Fig. 6a) and confirmed by RT-PCT (Fig. 6b). *XET* homologous gene from *P. euphratica* was selected for further analysis as *XET* gene regulated root growth was reported in pea^30^ and *Medicago truncatula* ^34^. Very recently similar results with different expression of XTH gene family was found in Arabidopsis plants roots subjected to drought stress by microarray determination^86^. A particularly tissue specific regulation of XTH genes contribute to loosening or strengthening the cell wall in well-defined topological organs/ regions of the plant contributing to tolerance/ susceptibility to water availability. The balance among cell wall hardening and loosening activities explains the parts of accelerated and decelerated root growth in the elongation zone^87^. Root tips in elongation zone are the main regions that produce specialized sorts of cells and tissues in a particularly pre-defined pattern to assist development during different moisture level^88^. In the case of high moisture level the expression was recorded high which showed a decrease expression trend by decreased moisture level, and the resulting *XET* activity was proposed to be involved in cell wall adaptation processes during root cell elongation, which might be stimulated by water gradients and thus shows hydrotropic growth. The previous studies showed that *XET* activity was boosted in the apical area of roots grown under low water potential^49,66,89^. These results indicated that the increased expression level of *XET* transcript in the root tips of Populus roots under various moisture levels might be necessary for maintaining root elongation under these conditions. The moderate water level provide better environment for high expression of Stress responsive signaling molecule and *XET* gene expression which showed positive result for root growth during drought stress. The differentially gene expression profile of *PeXET* gene of *P. euphratica* roots subjected to soil drying suggested that *PeXET* gene might be involve in the hydrotropic positive development of roots to absorb underground water. *PeXET* over-expression confirmed the increased drought tolerance and in this study and confirmed the involvement of *PeXET* gene in root development (Fig. 5). This data is supported by the work of Osata *et al*.^50^, by sorting out the Arabidopsis thaliana XTH genes are dominantly expressed in the roots. However, the expression of Arabidopsis thaliana XTH genes showed different trends and respond to hormonal signals differently. To investigate the *PeXET* gene functions in primary root growth, we examined phenotypes of loss-of-function mutants for these genes RNAi plants. These functional analyses disclosed a principal role for the *PeXET* gene in primary root elongation. Similar results for the four Arabidopsis thaliana XTH genes were confirmed by previous work^50^. The corresponding promoters region of *PeXET* was isolated and the bioinformatics analysis exposed the presence of growth regulation, growth and environment responsive element. Due to the presence of several cis-elements the expression of GUS gene was found higher in lower part as compared to leaves. The expression trend of PeXET promoter not only organ specific but also induced by external stimuli^63^.

Several other genes along with *PeXET* involved in the root response to soil drying (Fig. 6a). Further study should broaden our understanding of the molecular networks of the plant drought tolerance in a desert environment.

## Electronic supplementary material

Supplementary Information

Table S1 and S2

Table S3

Table S4
