# Supplementary material for: Physiological and transcriptome analysis of heteromorphic leaves and hydrophilic roots in response to soil drying in desert Populus euphratica
Source: Sci Rep. 2017 Sep 22;7:12188. doi: 10.1038/s41598-017-12091-2 (PMC5610244; doi:10.1038/s41598-017-12091-2)
Supplement: Supplementary file 1 — Supplementary Information [file 41598_2017_12091_MOESM1_ESM.doc]

Physiological and transcriptome analysis of heteromorphic leaves and hydrophilic roots in response to soil drying in desert *Populus euphratica*

Arshad Iqbal^a^, Tianxiang Wang^a^, Guodong Wu^a^, Wensi Tang^a^, Chen Zhu^a^, Dapeng Wang^a^, Yi Li ^b^, Huafang Wang^a^,*

^a^ *College of Biological Sciences and* [*Biotechnology*](app:ds:biotechnology)*, National Engineering Laboratory for Tree Breeding,* *Beijing Forestry University, Beijing 100083, China*

^b^ *Department of Plant Science, University of Connecticut, Storrs, CT 06269, USA*

* Corresponding author Tel./fax: +86 10 6233 8249; *E-mail address:*[hfwang@bjfu.edu.cn](mailto:hfwang@bjfu.edu.cn) (H. Wang)

Supplementary Figures


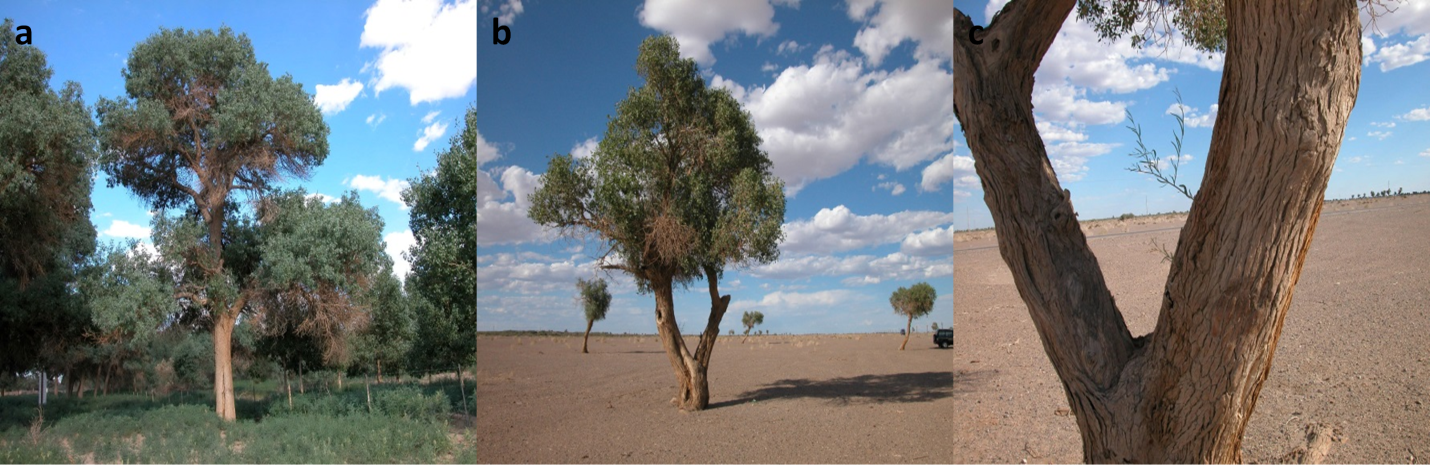


Supplementary Figure 1: Wide distribution of P. euphratica with diverse environmental condition. (a) Trees grow near water and have a wide trunk and a large crown (photo from desert river bank forest). (b-c): Trees from dry sparse woodland having narrow and dry trunks and display symptoms of being stressed.

Supplementary Figure 2: Genome structure of *PeXET* gene.


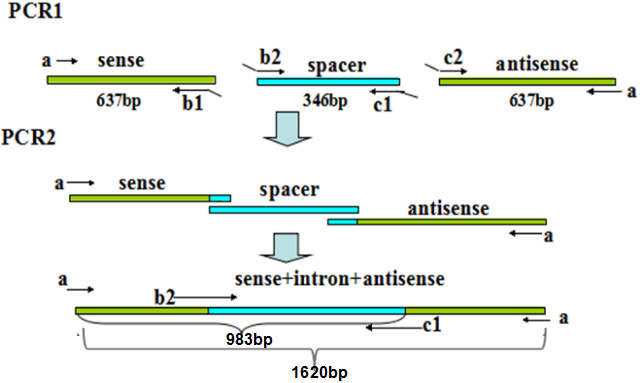


Supplementary Figure 3: Sketch of RNAi vector


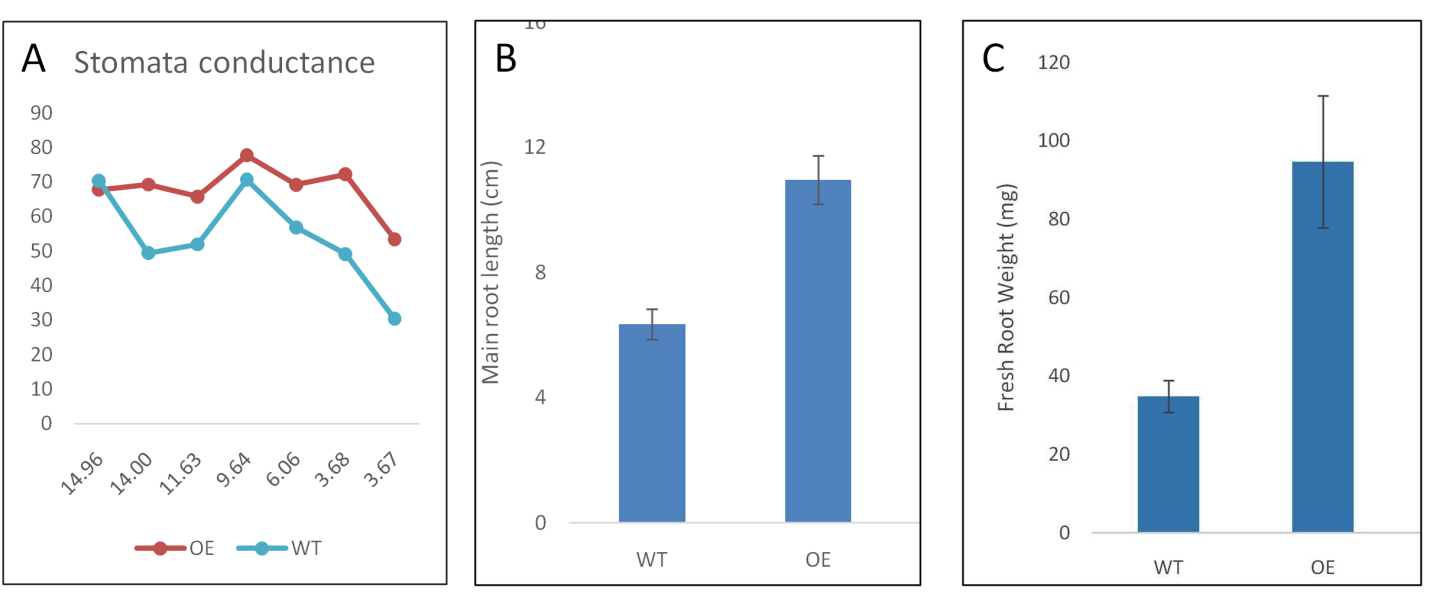


Supplementary Figure 4: Morphological comparison of *PeXET* transgenic OE tobacco and WT plants (n=5). (A)Stomata conductance of WT and T2 *PeXET* transgenic tobacco, (B) Main root length, (c) Fresh root weight
