# Supplementary material for: Physiological and transcriptome analysis of heteromorphic leaves and hydrophilic roots in response to soil drying in desert Populus euphratica
Source: Sci Rep. 2017 Sep 22;7:12188. doi: 10.1038/s41598-017-12091-2 (PMC5610244; doi:10.1038/s41598-017-12091-2)
Supplement: Supplementary file 2 — Table S1 and S2 [file 41598_2017_12091_MOESM2_ESM.doc]

Physiological and transcriptome analysis of heteromorphic leaves and hydrophilic roots in response to soil drying in desert *Populus euphratica*

Arshad Iqbal^a^, Tianxiang Wang^a^, Guodong Wu^a^, Wensi Tang^a^, Chen Zhu^a^, Dapeng Wang^a^, Yi Li ^b^, Huafang Wang^a^,*

^a^ *College of Biological Sciences and* [*Biotechnology*](app:ds:biotechnology)*, National Engineering Laboratory for Tree Breeding,* *Beijing Forestry University, Beijing 100083, China*

^b^ *Department of Plant Science, University of Connecticut, Storrs, CT 06269, USA*

* Corresponding author Tel./fax: +86 10 6233 8249; *E-mail address:*[hfwang@bjfu.edu.cn](mailto:hfwang@bjfu.edu.cn) (H. Wang)

Supplementary table 1: List of used primers

| **Primer name** | **Primer sequence** |
| --- | --- |
| **Plasmid Construction and tobacco molecular detection primer sequences** | |
| a | 5’-GTCTGTCTTCTCAAGTCCACCTC-3’ |
| b1 | 5’-GACAAGGCACTAGCGGGACAGTTATGGCTGCTTCTCTA-3’ |
| b2 | 5’-TAGAGAAGCAGCCATAACTGTCCCGCTAGTGCCTTGTC-3’ |
| c1 | 5’-ATAGAGAAGCAGCCATAACTCGAACGCCATTTGAAGCCGATGT-3’ |
| c2 | 5’-ACATCGGCTTCAAATGGCGTTCGAGTTATGGCTGCTTCTCTAT -3’ |
| kan-F | 5’-TTCGGCTATGACTGGGCACAACA-3’ |
| kan-R | 5’-ACTTTCTCGGCAGGAGCAAGGTG-3’ |
| XET-F | 5’-AAGTGAAGTTGGTTCCTGGTGAT-3’ |
| XET-R | 5’-CCATAGGACGGAGTAAGAGTGGT-3’ |
| **Primer sequences for PCR and RACE used for PeXET gene cloning** | |
| PeXET-722-1 | 5’-TCGAGATATGGCTGCTTCT-3’ |
| PeXET-999-3 | 5’-AAACAGGATGGAAGCAGCT-3’ |
| 3’ RACE/5’ RACE Oligo(dT)-adapter | 5’-GCGAGCACAGAATTAATACGACTCACTATAGG-oligo(dT)_12_ |
| 3’ RACE/5’ RACE Outer-Primer | 5’-CGAGCACAGAATTAATACGACTC-3’ |
| 3’ RACE/5’ RACE Inner-Primer | 5’-GAATTAATACGACTCACTATAGG-3’ |
| 3’ RACE Outer GSP | 5’-TGCCAATCAGAGTGTTCAAG-3’ |
| 3’ RACE Inner GSP | 5’-TGAAGTTGGTTCCTGGTGATT-3’ |
| 5’ RACE Outer GSP | 5’-GTGGCACAGAATTTCGCTT-3’ |
| 5’ RACE Inner GSP | 5’-TCATCCACGAAGAACACTAC-3’ |
| **Differential display RT-PCR** | |
| Anchor primer: |  |
| Cat G | 5 'AAG CTT TTT TTT TTT TTG 3' |
| Cat A | 5 'AAG CTT TTT TTT TTT TTA 3' |
| Cat C | 5 'AAG CTT TTT TTT TTT TTC 3' |
| Random primer: |  |
| 1 | 5 'AAG CTT GAT TGC C 3' |
| 2 | 5 'AAG CTT CGA CTG T 3' |
| 3 | 5 'AAG CTT TGG TCA G 3' |
| 4 | 5 'AAG CTT CTC AAC G 3' |
| 5 | 5 'AAG CTT AGT AGG C 3' |
| 6 | 5 'AAG CTT GCA CCA T 3' |
| 7 | 5 'AAG CTT AAC GAG G 3' |
| 8 | 5 'AAG CTT TTA CCG C 3' |
| **Populus Actin gene primer:** | |
| P1 | 5'-AACAGAAGAAACTTGGGT-3 ' |
| P2 | 5'-CTATCCAGGCAGTGCTCT-3 ' |
|  |  |
| **Primer used in cloning and identification of Populus PeXET** | |
| PeXET-iPCR11 | 5’- CTTTCTATGTAAGCGTTTCTC-3 ' |
| PeXET-iPCR12 | 5’- TATCAAACCTACTGCTGTTAG-3 ' |
| PeXET-iPCR21 | 5’-CCCTTTATCATCATTCCCA-3 ' |
| PeXET-iPCR22 | 5’-GAATCTCTGAGCCTCCATT-3 ' |
| iPCR2-11 | 5’-GATTGACGAAGCCCCACTAG-3 ' |
| iPCR2-12 | 5’-TTCTGGTGGCAGTGAAGGGT-3 ' |
| iPCR2-21 | 5’-TCCACACAGCTGCTTCCATC-3 ' |
| iPCR2-22 | 5’-CCCATCTGAGCCTCCTGTAT-3 ' |
| 1Up-HindIII-1831 | 5’-CCAAGCTTGGAAAGTGGTGATCGGTG-3 ' |
| 2Up-HindIII-1425 | 5’-CCAAGCTTCAGTTGATGGGTCGGGGTT-3 ' |
| 3Up-HindIII-993 | 5’-CCAAGCTTTTCCGGTTCATTGCACCAC-3 ' |
| 4Up-HindIII-717 | 5’-CCAAGCTTGACCCATTCACTTGCTTGC-3 ' |
| 5Up-HindIII-588 | 5’-CCAAGCTTCGTCACCGTAGTAGAGTCG-3 ' |
| p1301-Down-NcoI | 5’-TGCCATGGGTACTCCTGAAGGGGTGCT-3 ' |
|  |  |
| **Primer Used in RT-PCR validation** | |
| Carbonic anhydrase-F | AGGAGACAAGGCCCCATGACTGT |
| Carbonic anhydrase-R | GGATGACCCGGGAATCAGAGC |
| Bifunctional dihydroflavonol 4-reductase-F | CCACCGTCCGTGACCCTGAT |
| Bifunctional dihydroflavonol 4-reductase-R | CACACCCGTGCACCCTTGAAC |
| Ubiquitin-like protein 5-F | GACCGGCACCCGACCCGAT |
| Ubiquitin-like protein 5-R | GGCAAAGGAAGCATCGACTCGC |
| Protein phosphatase 2C-F | CCGATGGGTCTGTTCCACC |
| Protein phosphatase 2C-R | ACCACAAACAGAAGTCATGCCG |
| Mitogen-activated protein kinase kinase 2-F | CAAAGCGGCACGTTCCAGGAT |
| Mitogen-activated protein kinase kinase 2-R | GAGCAATGGCCCTGCGTGC |
| Histidine-containing phosphotransfer protein 5-F | GCCATTACGGCCGGGGAAGAG |
| Histidine-containing phosphotransfer protein 5-R | TTGCAGGGACCGGGAGTAGTTG |
| xyloglucan endotransglucosylase-F | CCAGGACCAGCAACCTGTGCTT |
| xyloglucan endotransglucosylase-R | ATACCGGCAACGCACTCTGGT |
| Ubiquitin-like protein 5-F | GACCGGCACCCGACCCGAT |
| Ubiquitin-like protein 5-R | GGCAAAGGAAGCATCGACTCGC |
| Inositol-3-phosphate synthase-F | TTATGCTTGTGGGGTGGGGCG |
| Inositol-3-phosphate synthase-R | TGGGGAGGAGGCTCTTGAATGG |
| Calmodulin-F | GCATCGCGAAAAGGCCTCGTG |
| Calmodulin-R | TGCCTGGAACTCCGACTCGAT |
| Glutathione S-transferase-F | TGGCTTTGTTGGGGCTTATCG |
| Glutathione S-transferase-R | CCAAGGGAGGCCCCATCGC |
| programmed cell death-F | CGGGGCAGGCAGATTCACAAT |
| programmed cell death-R | GCCTTCGCTCCTCAGCATCGTT |
| Leucoanthocyanidin dioxygenase-F | GGCCGGGGGCTTAGCGTTTC |
| Leucoanthocyanidin dioxygenase-R | AGCTCCTCCTGGGGCCGAAT |
| Glutathione S-transferase-F | TGGCTTTGTTGGGGCTTATCG |
| Glutathione S-transferase-R | CCAAGGGAGGCCCCATCGC |
| Transcription factor TGA1-F | GCAGTTGCAGAGACGGGAAAGT |
| Transcription factor TGA1-R | TCCACCACCCATATACAGGCCC |

**Supplemetray Table 2**: P. euphratica root DEGs trends in response to soil drying

T11: DEGs in 2.50% SWC vs control(3.50% SWC), T12: 2.59% SWC vs control(3.50% SWC), T13: 2.71% SWC vs control(3.50% SWC), T14: 2.79% SWC vs control(3.50% SWC).

| Unigene name | Pathway | Number of DEGs | | | | | | | |
| --- | --- | --- | --- | --- | --- | --- | --- | --- | --- |
|  |  | T14 | | T13 | | T12 | | T11 | |
|  |  | up | down | up | down | up | down | up | down |
| Inositol-3-phosphate synthase | Phospholipid signaling | 23 | - | 1 | 2 | - | 3 | - | - |
| Protein phosphatase 2C | Cell membrane signaling | - | - | 1 | - | 1 | - | - | 1 |
| MAPKK 2 | Cell membrane signaling | - | - | 1 | - | - | 2 | - | - |
| Calcium-binding protein | Cell membrane signaling | - | - | - | - | 1 | - | - | - |
| Histidine-containing phosphotransfer protein | Cytokinin signaling receptor | - | - | 1 | - | - | - | - | - |
| Homeobox-leucine zipper protein | Negative regulator of ABA signaling | 1 | - | 1 | - | - | - | - | - |
| Xyloglucan endotransglucosylase/hydrolase protein (XTH) | Cell wall biogenesis | 4 | - | 1 | 2 | 1 | 2 | - | - |
| Protein RALF-like | Cell expansion regulator | - | - | - | 1 | 4 | 1 | 3 | 1 |
| BI1-like protein | Cell death suppressor | 5 | - | - | 2 | - | - | - | - |
| Bark storage protein | Dormancy | 3 | 3 | - | 3 | 3 | - | - | 1 |
| Aquaporin | Water transportation | - | 2 | 7 | 1 | 2 | 2 | 1 | - |
| Myb transcription factor | Stress signal | 1 | - | 1 | - | - | - | 1 | - |
| NAC domain-containing protein | Development | 1 | - | 2 | - | - | - | - | - |
| Transcription factor TGA1 | Lateral root development | 1 | - | 1 | - | - | - | - | - |
| Remorin | Stress resistance | 1 | 1 | - | - | 2 | - | - | - |
| MADS-box transcription factor | Development | - | - | 1 | - | - | - | - | - |
| Auxin-responsive protein | Auxin signaling | - | - | - | - | 2 | - | 2 | - |
| SKP1-like protein | Protein degradation | 1 | - | - | - | - | - | - | - |
| BON1-associated protein | Cell death regulator | - | - | - | - | 1 | - | - | - |
